# Electronic structure, polaron formation, and functional properties in transition-metal tungstates

**DOI:** 10.1039/c7ra13436c

**Published:** 2018-01-23

**Authors:** Khang Hoang, Myungkeun Oh, Yongki Choi

**Affiliations:** a Department of Physics, North Dakota State University, Fargo, North Dakota 58108, USA. Email: yongki.choi@ndsu.edu; b Materials and Nanotechnology Program, North Dakota State University, Fargo, North Dakota 58105, USA

## Abstract

Transition-metal tungstates MWO_4_ (M = Co, Ni, Cu, Zn) have applications in many areas, including supercapacitors. A good understanding of the electronic structure is essential to understanding their functional properties. Here, we report a first-principles study of the materials using hybrid density-functional calculations. The electronic structure is analyzed with a focus on the nature of the electronic states near the band edges. We find that hole polarons can form at the Co lattice site in CoWO_4_ and the O site in NiWO_4_, CuWO_4_, and ZnWO_4_, resulting in the formation of Co^3+^ in the former and O^–^ in the latter. The electrochemical activity observed in certain tungstate compounds, but not in others, appears to be related to the ability to form hole polarons on the transition-metal ions. The formation energy and migration barrier of the hole polaron in CoWO_4_ are also calculated and the results are employed to understand the reported p-type conductivity.

## Abstract 2

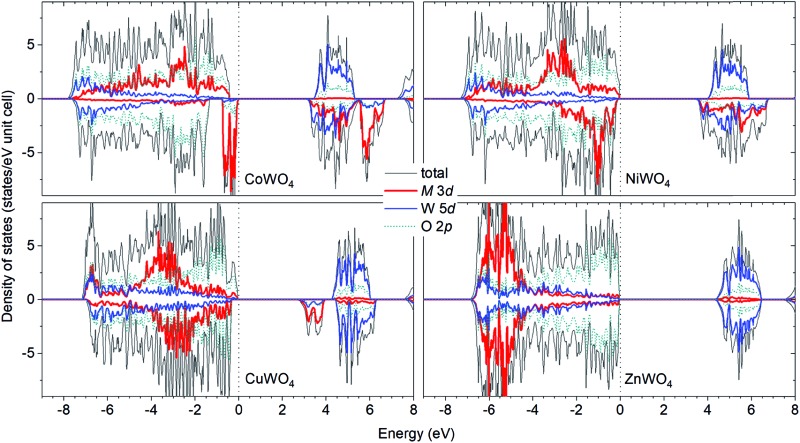
This work establishes a relationship between the electronic structure, polaron formation, and electrochemical activity in transition-metal tungstates.

## 1 Introduction

**Fig. 1:**
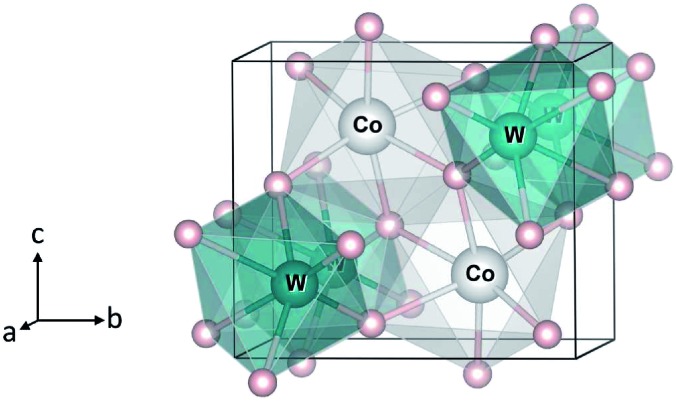
Crystal structure of monoclinic CoWO_4_. Large (gray) spheres are Co, medium (blue) spheres are W, and small (red) spheres are O.

Transition-metal tungstates MWO_4_ (M = Co, Ni, Cu, Zn) have been of interest for a wide range of applications, including supercapacitors,^1^,^2^ photocatalysts,^3^–^5^ scintillators,^6^ and sensors.^7^ The compounds possess the monoclinic (*P*2/*c*) wolframite-type structure,^8^–^10^ see Fig. 1, except CuWO_4_ whose symmetry is triclinically distorted with the space group *P*1 due to the Jahn–Teller effect associated with the Cu^2+^ ion.^10^ CoWO_4_, for example, were shown to be a promising supercapacitor electrode material,^1^ suggesting that the Co^3+/2+^ redox couple may be active. Bharati *et al.*^11^ also reported that CoWO_4_ is a “p-type semiconductor” and its electronic conduction is likely to occur through hopping of small polarons. ZnWO_4_, on the other hand, was found not to show any electroactivity when used as a pseudocapacitive electrode material.^12^ As shown in previous studies, electronic structure and polaron formation can provide crucial information on the electrochemical properties of complex transition-metal oxides.^13^–^15^

The electronic structure of the tungstates mentioned above has been investigated by several research groups using first-principles calculations based on density-functional theory (DFT) within the standard local-density or generalized gradient approximation and/or its extension DFT+*U* where *U* is the on-site Hubbard correction.^16^–^19^ The methods used in these previous studies are, however, often have limited predictive power because the electronic states in the materials (*e.g.*, the transition-metal d and oxygen p states) are not treated on equal footing. Besides, to our knowledge, polaron formation in the compounds has not been studied. Recently, two transition-metal tungstates, FeWO_4_ and MnWO_4_, have been studied in detail and the results show that both hole and electron polarons can occur and participate in charge transport and electrochemical processes.^15^ It thus remains to be explored if polarons also form in the other tungstates.

We herein report a first-principles study of MWO_4_ (M = Co, Ni, Cu, Zn) using a hybrid DFT/Hartree–Fock method in which all electronic states in the materials are treated on equal footing. The focus of this work is on the electronic structure, particularly the nature of the electronic states near the band edges, in the different tungstate compounds, and the ability to stabilize an electron and/or hole polaron. We also explicitly investigate the formation of selected intrinsic point defects in CoWO_4_ as well as the migration of hole polarons and discuss its relevance to the observed p-type electronic conduction. The electronic structure of FeWO_4_ and MnWO_4_ is also reproduced and included for comparison.

## 2 Methods

The total-energy calculations are based on DFT, using the Heyd–Scuseria–Ernzerhof (HSE06) screened hybrid functional,^20^ the projector augmented wave (PAW) method,^21^ and a plane-wave basis set, as implemented in the Vienna *Ab Initio* Simulation Package (VASP).^22^ The Hartree–Fock mixing parameter and the screening length are set to their standard values of 0.25 and 10 Å, respectively. We use the standard PAW potentials in the VASP database which treat Mn 3d^6^ 4s^1^, Fe 3d^7^ 4s^1^, Co 3d^8^ 4s^1^, Ni 3d^9^ 4s^1^, Cu 3d^10^ 4p^1^, Zn 3d^10^ 4p^2^, W 6s^2^ 5d^4^, and O 2s^2^ 2p^4^ explicitly as valence electrons and the rest as core electrons. The plane-wave basis-set cutoff is set to 500 eV and spin polarization is included. The calculations of bulk properties in MWO_4_ (two formula units per unit cell) are carried out using a 5 × 4 × 5 *k*-point mesh. Point defects in CoWO_4_ are modelled using 2 × 2 × 2 (96-atom) monoclinic supercells. Integrations over the supercell Brillouin zone is carried out using the *Γ* point. In all calculations, structural relaxations are performed with the HSE06 functional and the force threshold is chosen to be 0.01 eV Å^–1^.

The formation energy of a cobalt vacancy (*V*_Co_) with charge state *q* in CoWO_4_ is defined as

1 *E*^f^(*V*_Co_^*q*^) = *E*_tot_(*V*_Co_^*q*^) – *E*_tot_(bulk) + *μ*_Co_ + *q*(*E*_v_ + *μ*_e_) + *Δ*^*q*^,

where *E*_tot_(*V*_Co_^*q*^) and *E*_tot_(bulk) are, respectively, the total energy of a supercell containing the vacancy and that of an equivalent supercell of the perfect CoWO_4_. *μ*_Co_ is the atomic chemical potential of Co, referenced to the total energy per atom of the Co metal. *μ*_e_ is the electronic chemical potential, *i.e.*, the Fermi level, referenced to the valence-band maximum (VBM) in the bulk (*E*_v_). *Δ*^*q*^ is the correction term to align the electrostatic potentials of the bulk and defect supercells and to account for finite-size effects on the total energies of charged defects.^23^ The expression for the formation energy of a small hole polaron (*q* = +1) is similar to eqn (1), except that the atomic chemical potential term is zero since there is only the exchange of an electron with its reservoir. In eqn (1), vibrational contributions to the energies are neglected because they are small or negligible.^24^ Besides, significant cancellation occurs between different terms in the equation.

## 3 Results and discussion

### 3.1 Atomic and electronic structure

We start with the bulk properties of the tungstates. Fig. 1 shows the relaxed structure of monoclinic CoWO_4_; the structure of NiWO_4_ and ZnWO_4_ is similar. The lattice parameters of MWO_4_ are listed in Table 1. We find that the calculated values are in excellent agreement with the reported experimental ones.^8^–^10^

**Table 1:** Lattice parameters of tungstates MWO_4_ (M = Co, Ni, Cu, Zn)

	Experimental		Calculated
CoWO_4_	*a* = 4.659 Å	Ref. 8	*a* = 4.659 Å
	*b* = 5.667 Å		*b* = 5.716 Å
	*c* = 4.940 Å		*c* = 4.936 Å
	*β* = 89.94°		*β* = 89.95°
NiWO_4_	*a* = 4.599 Å	Ref. 9	*a* = 4.612 Å
	*b* = 5.660 Å		*b* = 5.675 Å
	*c* = 4.906 Å		*c* = 4.906 Å
	*β* = 90.03°		*β* = 89.92°
CuWO_4_	*a* = 4.7095 Å	Ref. 10	*a* = 4.772 Å
	*b* = 5.8452 Å		*b* = 5.935 Å
	*c* = 4.8885 Å		*c* = 4.871 Å
	*α* = 88.353°		*α* = 87.70°
	*β* = 92.508°		*β* = 93.27°
	*γ* = 97.205°		*γ* = 98.89°
ZnWO_4_	*a* = 4.6926 Å	Ref. 10	*a* = 4.702 Å
	*b* = 5.7213 Å		*b* = 5.755 Å
	*c* = 4.9281 Å		*c* = 4.917 Å
	*β* = 90.632°		*β* = 90.70°

In CoWO_4_, Co is found to be stable as high-spin Co^2+^ with a magnetic moment of 2.73 *μ*_B_; W is stable as W^6+^. Our calculations using 2 × 1 × 1 supercells of the unit cell shown in Fig. 1, similar to the 2 × 1 × 1 models for FeWO_4_ and MnWO_4_ described in ref. 15, indicate that the ferromagnetic (FM) and antiferromagnetic (AF) spin configurations are degenerate in energy.

**Fig. 2:**
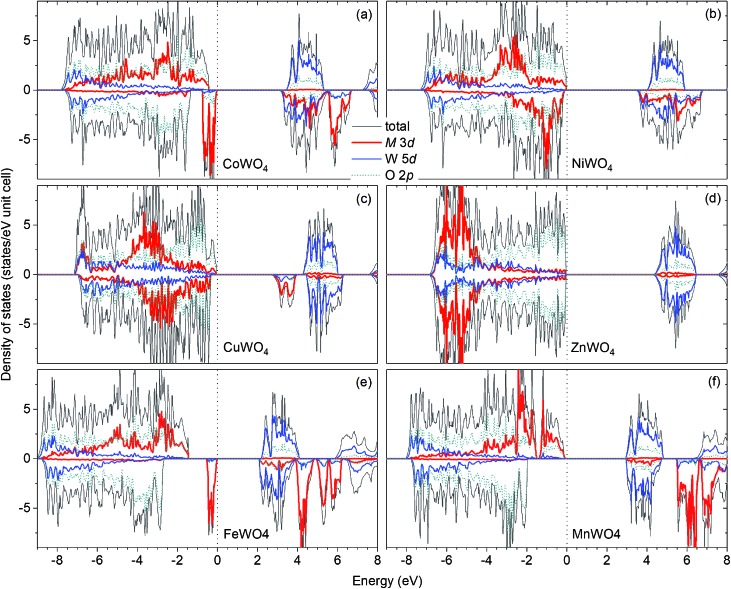
Total and projected electronic density of states of MWO_4_ in the ferromagnetic spin configuration: (a) M = Co, (b) M = Ni, (c) M = Cu, and (d) M = Zn. The results for (e) FeWO_4_ and (f) MnWO_4_ are also included for comparison. The zero of energy is set to the highest occupied states.

Fig. 2(a) shows the total and projected electronic density of states of CoWO_4_. Focusing on the electronic structure near the band edges, we find that the VBM is predominantly composed of the Co 3d states, whereas the conduction-band minimum (CBM) is predominantly the W 5d and Co 3d states. A detailed analysis of the electronic structure shows that each of the two Co atoms in the unit cell accounts for 39% of the electronic states at the VBM; at the CBM, each W accounts for 32% and each Co contributes 15%. The calculated band gap is 3.10 eV, a direct gap at the *Γ* point. This value is very close to the experimental value 2.80 eV estimated by Bharati *et al.*,^11^ obtained by assuming that the electronic conduction above 750 K is through band-like carriers.

As for NiWO_4_, Ni is stable as Ni^2+^ with a calculated magnetic moment of 1.74 *μ*_B_. The FM and AF spin configurations NiWO_4_ are almost degenerate in energy; the FM configuration is higher in energy than the AF configuration with parallel spins within the Ni zigzag chains along the *c* axis but with adjacent chains coupled antiferromagnetically by only 8 meV per formula unit. Fig. 2(b) shows the electronic structure of NiWO_4_. The VBM of the compound is predominantly composed of the Ni 3d states and the O 2p states; each Ni atom in the unit cell accounts for 16% of the electronic states at the VBM and some O atoms contribute up to 10% each. The CBM, on the other hand, is predominantly composed of the W 5d states and the Ni 3d states; each W atom accounts for 28% and each Ni atom contributes 20%. NiWO_4_ has an indirect band gap of 3.41 eV. Experimentally, the compound was reported to have an optical band gap of 3.2 ± 0.2 eV.^9^

In CuWO_4_, Cu is found to be stable as Cu^2+^ with a magnetic moment of 0.76 *μ*_B_. The electronic structure of the compound is shown in Fig. 2(c). We find that the VBM is predominantly composed of the O 2p states and some contribution from the Cu 3d states; specifically, the electronic states at the VBM have 12% from each Cu atom and some O atoms contributes up to 13% each. The CBM is, on the other hand, predominantly the Cu 3d states (29% from each Cu atom) and the W 5d states (17% from each W atom). CuWO_4_ is found to have an indirect band gap of 2.71 eV, comparable to the reported experimental value 2.3 eV.^4^

Finally, Zn in ZnWO_4_ is found to be stable as Zn^2+^ with a zero magnetic moment. Fig. 2(d) shows the electronic structure of ZnWO_4_. We find that the VBM is predominantly composed of the O 2ps states; some O atoms contribute up to 20% to the electronic states at the VBM. The CBM is, on the other hand, predominantly the W 5d states (each W atom contributes 40%) and there is a small contribution from the Zn 3d states (5% is from each Zn atom). ZnWO_4_ has a calculated (direct) band gap of 4.30 eV, comparable to the reported experimental value 3.98 eV.^16^

For comparison, the results for FeWO_4_ and MnWO_4_ are also included; see Fig. 2(e and f). As also reported ref. 15, the VBM of FeWO_4_ is predominantly composed of the Fe 3d states with each Fe atom accounts for 46% of the electronic states at the VBM; the CBM is predominantly the W 5d states with each W atom contributes 40%. The VBM of MnWO_4_ is, on the other hand, predominantly the Mn 3d states with each Mn atom accounts for 27%; the CBM is predominantly composed of the W 5d states with each W atom accounts for 39% of the states at the CBM.

The nature of the electronic structure near the band edges is thus different for different tungstates in the series, as expected. More importantly, our analysis provides a quantitative understanding of electronic structure formation. In the next section, we will examine the implications on defect formation in the materials, particularly on possible formation of polarons.

### 3.2 Hole polaron formation

Calculations for polarons are carried out using the supercell models described in Section 2. For each compound, the creation of a free hole polaron involves removing one electron from the supercell, specifically from the highest occupied state; the creation of a free electron polaron, on the other hand, involves adding one electron to the supercell, *i.e.*, to the lowest unoccupied state. The procedures are performed *via* controlling the total number of valence electrons in the system. The actual occupation of the removed or added electron is checked by examining changes in the charge density, local lattice environment, and magnetic moment. The removal and addition of electrons can be employed to represent oxidation and reduction processes, respectively.^13^–^15^

**Fig. 3:**
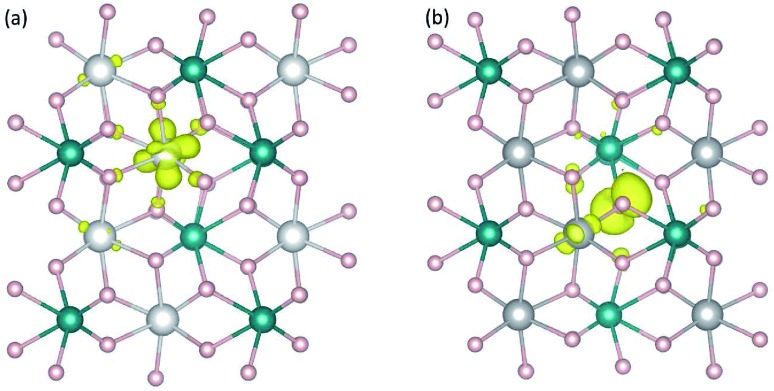
Charge densities associated with hole polaron (a) *η*_Co_^+^ in CoWO_4_ and (b) *η*_O_^+^ in NiWO_4_. The isovalue for the isosurface (yellow) is set to 0.05 *e*/Å^3^. Large (gray) spheres are Co or Ni, medium (blue) spheres are W, and small (red) spheres are O.

We find that the removal of an electron from the CoWO_4_ supercell results in a high-spin Co^3+^ with a calculated magnetic moment of 3.16 *μ*_B_, which can be regarded as a highly localized electron hole at one of the Co lattice sites. The local lattice environment is slightly distorted in the presence of the hole; the average Co^3+^–O bond length is 2.02 Å, compared to 2.10 Å of the Co–O bonds in the bulk. Since the lattice distortion is mainly within the first nearest neighbors of the hole, the localized hole can be regarded as a small hole polaron, hereafter denoted as *η*_Co_^+^; see Fig. 3(a). The self-trapping energy (*E*_ST_), defined as the formation-energy difference between the free hole and the hole polaron, is calculated to be 0.32 eV for *η*_Co_^+^. We note that the low-spin Co^3+^ (0 *μ*_B_) solution is higher in energy than the high-spin configuration by 0.50 eV. The formation of the hole polaron *η*_Co_^+^ in CoWO_4_ can be understood in terms of the electronic structure discussed earlier according to which the electron has to be removed from the Co 3d states; see also Fig. 2(a).

The removal of an electron from the NiWO_4_, CuWO_4_, or ZnWO_4_ supercell, on the other hand, results in a highly localized electron hole at one of the O sites; *i.e.*, one O^2–^ is oxidized to O^–^ with a magnetic moment of ∼0.7 *μ*_B_. The local lattice environment is also distorted in the presence of this hole. O^–^ can thus also be regarded as a small hole polaron, hereafter denoted as *η*_O_^+^. Fig. 3(b) shows the charge density associated with *η*_O_^+^ in NiWO_4_. The self-trapping energy is *E*_ST_ = 0.22 eV (in NiWO_4_), 0.05 eV (CuWO_4_), or 0.38 eV (ZnWO_4_). The very small *E*_ST_ value in the case of triclinic CuWO_4_ could be due to the Jahn–Teller distortion in the material's lattice environment. The main difference between these three tungstates and CoWO_4_ (as well as FeWO_4_ and MnWO_4_)^15^ is thus that the oxidation occurs on the transition metal in the latter whereas it can occur on the oxygen in the former. The results are therefore consistent with the electronic structure presented earlier in which the VBM of NiWO_4_, CuWO_4_, and ZnWO_4_ is composed mainly of the O 2p states. We note that, though a free hole polaron associated with Ni^3+^ cannot be stabilized in bulk NiWO_4_, it remains to be explored if it can occur in the presence of other defects or at/near the surface or interface where the lattice environment is different from that in the bulk.

Regarding electron polarons, we find that the addition of an electron to MWO_4_ (M = Co, Ni, Cu, Zn) results in an electron that is delocalized all over the supercell. The electron polaron associated with the reduction of W^6+^ to W^5+^ is thus not stable in these compounds, unlike in FeWO_4_ and MnWO_4_.^15^ The difference can be traced back to the electronic structure discussed earlier: there is a strong mixing between the M 3d and W 5d states at the CBM of MWO_4_ (M = Co, Ni, Cu, Zn). As a result, an electron when added to the materials (*e.g.*, during a reduction process) cannot be localized on any particular W ion. In the case of MWO_4_ (M = Fe, Mn), the CBM is predominantly composed of the W 5d states, making it possible for the added electron to be localized.^15^

Finally, as mentioned earlier, some transition-metal tungstates such as CoWO_4_ (as well as FeWO_4_ and MnWO_4_) are electroactive,^1^,^12^,^25^ whereas other tungstates such as ZnWO_4_ are not active,^12^ when used as pseudocapacitive electrode materials. This can be ascribed to the fact that hole polarons associated with the transition metal can form in the former but not in the latter; *i.e.*, the transition-metal (M) redox couple is active in MWO_4_ (M = Co, Fe, Mn). Given the interplay between the electronic structure and polaron formation, our work thus illustrates how a material's functional properties can be related to its electronic structure.

### 3.3 Electronic conduction in CoWO_4_

**Fig. 4:**
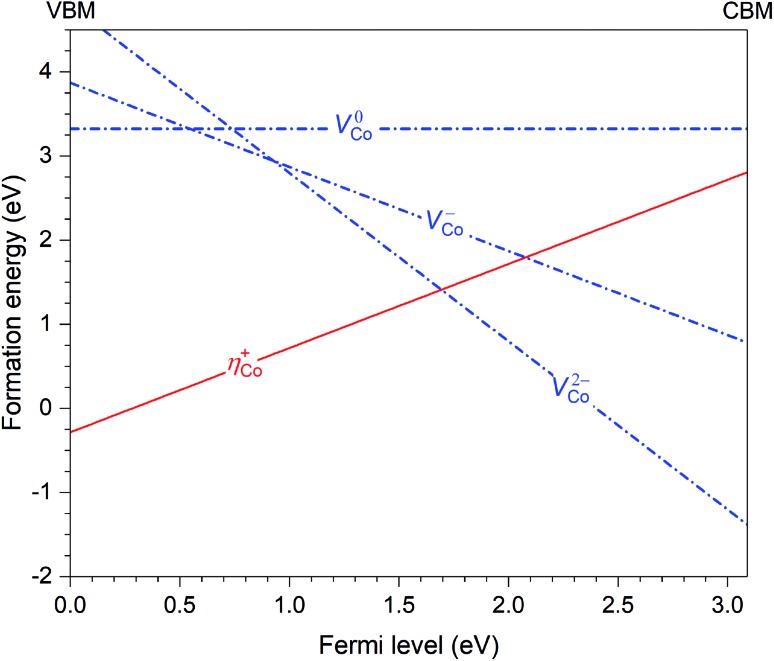
Formation energies of the hole polaron (*η*_Co_^+^) and Co vacancies (*V*_Co_) in CoWO_4_, plotted as a function of Fermi level from the valence-band maximum (VBM) to the conduction-band minimum (CBM).

Let us now discuss in more detail the case of CoWO_4_ where the hole polaron *η*_Co_^+^ is found to be stable. This defect can occur in the material in combination with other intrinsic point defects. Based on the results previously reported for FeWO_4_ and MnWO_4_,^15^ we consider only cobalt vacancies (*V*_Co_) since *V*_Co_^2–^ (*i.e.*, the removal of an Co^2+^ ion) is likely the lowest-energy negatively charged intrinsic point defect. Fig. 4 shows the calculated formation energies of *η*_Co_^+^ and *V*_Co_ (in charge states 2–, –, and 0). The slope in the energy plots indicates the charge state: positively (negatively) charged defects have positive (negative) slopes. The formation energies are obtained by assuming that CoWO_4_ is in equilibrium with WO_3_ (often used as one of the reactants in the synthesis of CoWO_4_) and air at 900 °C (the conditions under which the material is prepared).^17^ We note that the *q* = –1 and 0 charge states of *V*_Co_, nominally denoted as *V*_Co_^–^ and *V*_Co_^0^, are not really stable; *V*_Co_^–^ (*V*0Co) is, in fact, a defect complex of *V*_Co_^2–^ and one (two) *η*_Co_^+^.

Since *η*_Co_^+^ and *V*_Co_^2–^ are the dominant intrinsic point defects in the material, assuming the absence of any lower-energy negatively charged intrinsic defects or impurities, they determine the position of the Fermi level, which is at *μ*inte = 1.67 eV, where charge neutrality is maintained; see Fig. 4. At this Fermi-level position, the formation energy of the two defects is 1.41 eV. We also estimate the migration barrier (*E*_m_) of the hole polaron, using the method as described in ref. 15 and references therein, and find that *E*_m_ = 0.29 eV along the zigzag metal chain (*i.e.*, *c* axis). For comparison, the migration barrier was reported to be 0.14 eV for *η*_Fe_^+^ in FeWO_4_ or 0.28 eV for *η*_Mn_^+^ in MnWO_4_.^15^

Given the low migration barrier, *η*_Co_^+^ can participate in electronic transport that results in the p-type conductivity as observed in experiments.^11^ Bharati *et al.* reported that the activation energy in the extrinsic region (<750 K) is *E*_a_ = 0.64 eV, whereas *E*_a_ = 1.40 eV in the intrinsic region (>750 K). As discussed in detail in ref. 15 and 26, the lower limit of the activation energy is *E*_m_ and the upper limit is *E*^f^ + *E*_m_. The calculated *E*_m_ is thus lower than the experimental *E*_a_ in the extrinsic region, as expected. In the intrinsic region, our calculations give *E*_a_ = 1.70 eV. Experimentally, Bharati *et al.* argued that the conduction is band-type with an activation energy of 1.40 eV;^11^ however, given that our estimated activation energy is comparable, the conduction may actually still involve hopping of the small hole polarons *η*_Co_^+^.

## 4 Summary

A hybrid density-functional study of the electronic structure and polaron formation has been carried out for transition-metal tungstates. The calculated lattice parameters and band gaps are in good agreement with experiments. The nature of the electronic structure at the valence-band top in CoWO_4_ allows for the formation of hole polarons (Co^3+^) at the transition metal site and hence active (Co^3+/2+^) redox couples, similar to what was reported for FeWO_4_ and MnWO_4_, whereas in NiWO_4_, CuWO_4_, and ZnWO_4_ hole polarons (O^–^) can be stabilized at the oxygen site. Electron polarons at the W site cannot be stabilized in MWO_4_ (M = Co, Ni, Cu, Zn), unlike in FeWO_4_ and MnWO_4_ where the electron polarons were reported to be stable. The difference between the tungstate compounds can be understood in terms of the calculated electronic structure. Finally, we find that hole polarons are responsible for the observed p-type conductivity in CoWO_4_.

## Conflicts of interest

There are no conflicts to declare.
